# Pair correlation and twin primes revisited

**DOI:** 10.1098/rspa.2016.0548

**Published:** 2016-10

**Authors:** Brian Conrey, Jonathan P. Keating

**Affiliations:** 1 American Institute of Mathematics, 600 East Brokaw Road, San Jose, CA 95112, USA; 2 School of Mathematics, University of Bristol, Bristol BS8 1TW, UK

**Keywords:** pair correlation, random matrix theory, twin primes

## Abstract

We establish a connection between the conjectural two-over-two ratios formula for the Riemann zeta-function and a conjecture concerning correlations of a certain arithmetic function. Specifically, we prove that the ratios conjecture and the arithmetic correlations conjecture imply the same result. This casts a new light on the underpinnings of the ratios conjecture, which previously had been motivated by analogy with formulae in random matrix theory and by a heuristic recipe.

## 1. Introduction

Montgomery in his famous pair correlation paper [1] used heuristics based on the Hardy–Littlewood conjecture concerning the distribution of prime pairs [2] to conclude that pairs of zeros of the Riemann zeta-function have the same scaled statistics, in the limit in which their height up the critical tends to infinity, as pairs of eigenvalues of large random Hermitian matrices (or of unitary matrices with Haar measure). Montgomery did not give the details of the calculation involving twin primes in his paper, but that calculation has been repeated with variations several times in the literature (e.g. [3–7]). Goldston & Montgomery [8] proved rigorously that the pair correlation conjecture is equivalent to an asymptotic formula for the variance of the number of primes in short intervals, and Montgomery & Soundararajan [9] proved that this variance formula follows from the Hardy–Littlewood prime-pair conjecture, under certain assumptions.

In a slightly different vein, Bogomolny & Keating [10,11] and later Conrey & Snaith [12] developed methods to give more precise estimates for the pair correlation (and higher correlations) of Riemann zeros. Bogomolny and Keating gave four different heuristic methods to accomplish this, while Conrey and Snaith used a uniform version of what is known as the ratios conjecture from which assumption they could rigorously derive this precise form of pair correlation. All of these methods lead to the same formulae.

In this paper, we reconsider this circle of ideas from yet another perspective, namely that of deriving a form of the ratios conjecture from consideration of correlations between the values of a certain arithmetic function. This provides a new perspective on the underpinnings of the ratios conjecture, which previously had been motivated by analogy with formulae in random matrix theory and by a heuristic recipe [13–15]. This is similar to how, in a recent series of papers [16–19] we have shown that moment conjectures previously developed using random matrix theory [14,20] may be recovered from correlations of divisor sums.

The twin prime conjectures are easily stated in terms of the von Mangoldt function *Λ*(*n*) which is the generating function for −*ζ*′/*ζ* (e.g. [21]):

$$-\frac{\zeta^{\prime }}{\zeta }(s)=\sum_{n=1}^{\infty }\frac{\Lambda (n)}{n^{s}}$$

or equivalently

$$\Lambda (n)=\left\{ \begin{array}{ll} \log p & \mathrm{if}\;n=p^{k}\;\mathrm{for}\;\mathrm{some}\;\mathrm{prime}\;p\\ 0 & \mathrm{otherwise}. \end{array}\right.$$

In the Conrey–Snaith approach, zeros of *ζ*(*s*) are detected as poles of (*ζ*′/*ζ*)(*s*) which in turn is realized via

ζ′ζ(s)=ddαζ(s+α)ζ(s+γ)|α=0γ=0.

Passing to coefficients, we write

$$I_{\alpha ,\gamma }(s)=\sum_{n=1}^{\infty }\frac{I_{\alpha ,\gamma }(n)}{n^{s}}=\frac{\zeta (s+\alpha )}{\zeta (s+\gamma )};$$

explicitly

$$I_{\alpha ,\gamma }(n)=\sum_{de=n}\frac{\mu (e)}{d^{\alpha }e^{\gamma }}.$$

Note that

$$I_{\alpha ,\gamma }(n)=n^{r}I_{\alpha +r,\gamma +r}(n)$$

for any *r*. Also we have

Λ(n)=−ddαIα,γ(n)|α=0γ=0.

Here we will investigate the averages

$$R_{\alpha ,\beta ,\gamma ,\delta }(T):=\int_{0}^{\infty }\psi \left(\frac{t}{T}\right)\frac{\zeta (s+\alpha )\zeta (1-s+\beta )}{\zeta (s+\gamma )\zeta (1-s+\delta )}\;dt,$$

where $s=\frac{1}{2}+it$ and *ψ*(*z*) is holomorphic in a strip around the real axis and decreases rapidly on the real axis. Not surprisingly, R is related to averages of the (analytic continuation of the) Rankin–Selberg convolution

$$B_{\alpha ,\beta ,\gamma ,\delta }(s):=\sum_{n=1}^{\infty }\frac{I_{\alpha ,\gamma }(n)I_{\beta ,\delta }(n)}{n^{s}}.$$

In fact, the simplest case of the ratios conjecture asserts that

1.1 $$R_{\alpha ,\beta ,\gamma ,\delta }(T)=\int_{0}^{\infty }\psi \left(\frac{t}{T}\right)\left(B_{\alpha ,\beta \gamma ,\delta }(1)+{\left(\frac{t}{2\pi }\right)}^{-\alpha -\beta }B_{-\beta ,-\alpha ,\gamma ,\delta }(1)\right)dt+O(T^{1-\eta })$$

for some *η*>0. It is also not surprising that R is connected to weighted averages over *n* and *h* of

$$I_{\alpha ,\gamma }(n)I_{\beta ,\delta }(n+h).$$

It is this connection that we are elucidating. Using the *δ*-method, it transpires that these weighted averages may be expressed in terms of

$$\begin{array}{rl} C_{\alpha ,\beta ,\gamma ,\delta }(s) & :=\frac{1}{{\left(2\pi i\right)}^{2}}\int_{|w-1|=\epsilon }\int_{|z-1|=\epsilon }\chi (w+z-s-1)\sum_{q=1}^{\infty }\sum_{h=1}^{\infty }\frac{r_{q}(h)}{h^{s+2-w-z}}\\ & \;\times \sum_{m=1}^{\infty }\frac{I_{\alpha ,\gamma }(m)e(m/q)}{m^{w}}\sum_{n=1}^{\infty }\frac{I_{\gamma ,\delta }(n)e(n/q)}{n^{z}}\;dw\;dz, \end{array}$$

where *r*_*q*_(*h*) denotes Ramanujan’s sum and where *χ*(*s*) is the factor from the functional equation *ζ*(*s*)=*χ*(*s*)*ζ*(1−*s*); also here and elsewhere *ϵ* is chosen to be larger than the absolute values of the shift parameters *α*,*β*,*γ*,*δ* but smaller than $\frac{1}{2}$. The result that ties this all together is the following identity.

**Theorem 1.1**

*Assuming the generalized Riemann hypothesis
*

$$C_{\alpha ,\beta ,\gamma ,\delta }(s)=B_{-\beta ,-\alpha ,\gamma ,\delta }(s+1).$$

In a recent series of papers [16–19], we have outlined a method that involves convolutions of coefficient correlations and leads to conclusions for averages of truncations of products of shifted zeta-functions implied by the recipe of [14]. In this paper, we strike out in a new direction, using similar ideas to evaluate averages of truncations of products of ratios of shifted zeta-functions. In particular, the approach of Bogolmony & Keating [6,7] on convolutions of shifted coefficient sums guide the calculations and we are led, as in the previous series, to formulate a kind of multi-dimensional Hardy–Littlewood circle method. This first paper, as indicated above, may be viewed in a more classical context.

It turns out to be convenient to study an average of the ratios conjecture. To this end, let

$$I_{\alpha ,\gamma }(s;X)=\sum_{n\leq X}I_{\alpha ,\gamma }(n)n^{-s}.$$

We are interested in the average over *t* of $I_{\alpha ,\gamma }{\bar{I}}_{\beta ,\delta }$ in the case that *X*=*T*^λ^ for some λ>1. (When λ<1 this average is dominated by diagonal terms.) We give two different treatments of the average of ‘truncated’ ratios:

$$M_{\alpha ,\beta ,\gamma ,\delta }(T;X):=\int_{0}^{\infty }\psi \left(\frac{t}{T}\right)I_{\alpha ,\gamma }(s,X)I_{\beta ,\delta }(1-s,X)\;dt,$$

(where again *s*=1/2+*it*) which lead to the same answer. The first is by the ratios conjecture and the second is by consideration of the correlations of the coefficients.

In each case, we prove the following theorem.

**Theorem 1.2**

*Let α,β,γ,δ be complex numbers smaller than* 1/4 *in absolute value. Then, assuming either a uniform version of the ratios conjecture or a uniform version of a conjectured formula for correlations of values of I*_*α*,*γ*_(*n*) (*conjecture5.1, §5*), *we have for some η*>0 *and some* λ>1,

$$\begin{array}{rl} & M_{\alpha ,\beta ,\gamma ,\delta }(T;X)\\ & \;=\int_{0}^{\infty }\psi \left(\frac{t}{T}\right)\frac{1}{2\pi i}\int_{ℜs=2}\left(B_{\alpha ,\beta ,\gamma ,\delta }(s+1)+{\left(\frac{t}{2\pi }\right)}^{-\alpha -\beta -s}B_{-\beta ,-\alpha ,\gamma ,\delta }(s+1)\right)\frac{X^{s}}{s}\;ds\;dt+O(T^{1-\eta }). \end{array}$$

This shows that the ratios conjecture follows not only from the ‘recipe’ of [14,15], but also relates to correlations of values of *I*_*α*,*γ*_(*n*).

## 2. Approach via the ratios conjecture

We have

$$I_{\alpha ,\gamma }(s,X)=\frac{1}{2\pi i}\int_{\left(2\right)}I_{\alpha ,\gamma }(s+w)\frac{X^{w}}{w}\;dw;$$

there is a similar expression for $I_{\beta ,\delta }(s,X)$. Inserting these expressions and rearranging the integrations, we have

$$M_{\alpha ,\beta ,\gamma ,\delta }(T;X)=\frac{1}{{\left(2\pi i\right)}^{2}}\int_{ℜw=2}\int_{ℜz=2}\frac{X^{w+z}}{wz}R_{\alpha +w,\beta +z,\gamma +w,\delta +z}(T)\;dw\;dz.$$

We observe from expression (1.1) for the ratios conjecture that the integrand $R_{\alpha +w,\beta +z,\gamma +w,\delta +z}$ is, to leading order in *T*, expected to be a function of *z*+*w*. We therefore make the change of variable *s*=*z*+*w*; now the integration in the *s* variable is on the vertical line ℜ*s*=4. We retain *z* as our other variable and integrate over it. This turns out to be the integral

$$\frac{1}{2\pi i}\int_{ℜz=2}\frac{dz}{z(s-z)}=\frac{1}{s}$$

as is seen by moving the path of integration to the left to ℜz=−∞. Thus, we have that $M_{\alpha ,\beta ,\gamma ,\delta }(T;X)$ is given to leading order by

$$\frac{1}{2\pi i}\int_{ℜs=4}\frac{X^{s}}{s}R_{\alpha +s,\beta ,\gamma +s,\delta }(T)\;ds.$$

We move the path of integration to ℜ*s*=*ϵ*, avoiding crossing any poles, insert the ratios conjecture (1.1) (cf. the uniform version as laid out in [12]), and observe that

$$B_{\alpha +s,\beta ,\gamma +s,\delta }(1)=B_{\alpha ,\beta ,\gamma ,\delta }(s+1).$$

In this way, we have that the uniform ratios conjecture implies the conclusion of theorem 1.2.

## 3. Approach via coefficient correlations

We follow the methodology developed by Goldston & Gonek [5] on mean-values of long Dirichlet polynomials.

If we expand the sums and integrate term-by-term, we have

$$M_{\alpha ,\beta ,\gamma ,\delta }(T;X)=T\sum_{m,n\leq X}\frac{I_{\alpha ,\gamma }(m)I_{\beta ,\delta }(n)}{\sqrt{mn}}\hat{\psi }\left(\frac{T}{2\pi }\log\frac{m}{n}\right).$$

### (a) Diagonal

The diagonal term is

$$T\hat{\psi }(0)\sum_{m\leq X}\frac{I_{\alpha ,\gamma }(m)I_{\beta ,\delta }(m)}{m}.$$

By Perron’s formula, the sum here is

$$\frac{1}{2\pi i}\int_{\left(2\right)}B_{\alpha ,\beta ,\gamma ,\delta }(s+1)\frac{X^{s}}{s}\;ds.$$

### (b) Off-diagonal

For the off-diagonal terms, we need to analyse

$$2T\sum_{T\leq m\leq X}\sum_{1\leq h\leq X/T}\frac{I_{\alpha ,\gamma }(m)I_{\beta ,\delta }(m+h)}{m}\hat{\psi }\left(\frac{Th}{2\pi m}\right).$$

We replace the arithmetic terms by their average and express this as

$$2T\int_{T}^{X}\sum_{1\leq h\leq X/T}\frac{{\left\langle I_{\alpha ,\gamma }(m)I_{\beta ,\delta }(m+h)\right\rangle }_{m∼u}}{u}\hat{\psi }\left(\frac{Th}{2\pi u}\right)du.$$

We compute the average heuristically via the delta-method [22]:

$${\left\langle I_{\alpha ,\gamma }(m)I_{\beta ,\delta }(m+h)\right\rangle }_{m∼u}∼\sum_{q=1}^{\infty }r_{q}(h){\left\langle I_{\alpha ,\gamma }(m)e\left(\frac{m}{q}\right)\right\rangle }_{m∼u}{\left\langle I_{\beta ,\delta }(m)e\left(\frac{m}{q}\right)\right\rangle }_{m∼u},$$

where *r*_*q*_(*h*) is the Ramanujan sum, a formula for which is $r_{q}(h)=\sum_{\begin{array}{c} d|h\\ d|q \end{array}}d\mu (q/d)$; note that to actually prove this formula would be as difficult as proving the Twin Prime conjecture. We formalize this as a precise conjecture in §5. It is this conjecture that we refer to in theorem 1.2. Now

$${\left\langle I_{\alpha ,\gamma }(m)e\left(\frac{m}{q}\right)\right\rangle }_{m∼u}=\frac{1}{2\pi i}\int_{|w-1|=\epsilon }\sum_{m=1}^{\infty }I_{\alpha ,\gamma }(m)e\left(\frac{m}{q}\right)m^{-w}u^{w-1}\;dw.$$

Thus, the off-diagonal contribution is

$$\begin{array}{rl} & 2T\sum_{1\leq h\leq X/T}\int_{T}^{X}\frac{1}{{\left(2\pi i\right)}^{2}}\iint_{\begin{array}{c} |w-1|=\epsilon \\ |z-1|=\epsilon \end{array}}\sum_{q=1}^{\infty }r_{q}(h)\hat{\psi }\left(\frac{Th}{2\pi u}\right)u^{w+z-2}\\ & \;\times \sum_{m_{1}=1}^{\infty }\frac{I_{\alpha ,\gamma }(m_{1})e(m_{1}/q)}{m_{1}^{w}}\sum_{m_{2}=1}^{\infty }\frac{I_{\beta ,\delta }(m_{2})e(m_{2}/q)}{m_{2}^{z}}\;dw\;dz\frac{du}{u}. \end{array}$$

We make the change of variables *v*=*Th*/2*πu*. The inequality *u*≤*X* then implies that *Th*/2*πv*≤*X* or *h*≤2*πvX*/*T*. The above can be re-expressed as

$$\begin{array}{rl} & 2T\int_{0}^{\infty }\sum_{1\leq h\leq 2\pi vX/T}\frac{1}{{\left(2\pi i\right)}^{2}}\iint_{\begin{array}{c} |w-1|=\epsilon \\ |z-1|=\epsilon \end{array}}\sum_{q=1}^{\infty }r_{q}(h)\hat{\psi }(v){\left(\frac{Th}{2\pi v}\right)}^{w+z-2}\\ & \;\times \sum_{m_{1}=1}^{\infty }\frac{I_{\alpha ,\gamma }(m_{1})e(m_{1}/q)}{m_{1}^{w}}\sum_{m_{2}=1}^{\infty }\frac{I_{\beta ,\delta }(m_{2})e(m_{2}/q)}{m_{2}^{z}}dw\;dz\frac{dv}{v}. \end{array}$$

Using Perron’s formula to capture, the sum over *h* gives

$$\begin{array}{rl} & 2T\int_{0}^{\infty }\frac{1}{{\left(2\pi i\right)}^{3}}\int_{ℜs=2}\iint_{\begin{array}{c} |w-1|=\epsilon \\ |z-1|=\epsilon \end{array}}\sum_{q=1}^{\infty }\sum_{h=1}^{\infty }\frac{r_{q}(h)}{h^{s}}\hat{\psi }(v){\left(\frac{Th}{2\pi v}\right)}^{w+z-2}{\left(\frac{2\pi vX}{T}\right)}^{s}\\ & \;\times \sum_{m_{1}=1}^{\infty }\frac{I_{\alpha ,\gamma }(m_{1})e(m_{1}/q)}{m_{1}^{w}}\sum_{m_{2}=1}^{\infty }\frac{I_{\beta ,\delta }(m_{2})e(m_{2}/q)}{m_{2}^{z}}\frac{ds}{s}\;dw\;dz\frac{dv}{v}. \end{array}$$

Now

$$2\int_{0}^{\infty }\hat{\psi }(v)v^{A}\frac{dv}{v}=\chi (1-A)\int_{0}^{\infty }\psi (t)t^{-A}\;dt.$$

Incorporating this formula leads us to

$$\begin{array}{rl} & T\int_{0}^{\infty }\psi (t)\frac{1}{{\left(2\pi i\right)}^{3}}\int_{ℜs=2}\iint_{\begin{array}{c} |w-1|=\epsilon \\ |z-1|=\epsilon \end{array}}\sum_{q=1}^{\infty }\sum_{h=1}^{\infty }\frac{r_{q}(h)}{h^{s+2-w-z}}{\left(\frac{Tt}{2\pi }\right)}^{w+z-2}{\left(\frac{2\pi X}{tT}\right)}^{s}\chi (w+z-s-1)\\ & \;\times \sum_{m_{1}=1}^{\infty }\frac{I_{\alpha ,\gamma }(m_{1})e(m_{1}/q)}{m_{1}^{w}}\sum_{m_{2}=1}^{\infty }\frac{I_{\beta ,\delta }(m_{2})e(m_{2}/q)}{m_{2}^{z}}\frac{ds}{s}\;dw\;dz\;dt. \end{array}$$

Hence, by theorem 1.1, this is

$$\int_{0}^{\infty }\psi \left(\frac{t}{T}\right)\frac{1}{2\pi i}\int_{ℜs=2}{\left(\frac{t}{2\pi }\right)}^{-\alpha -\beta -s}B_{-\beta ,-\alpha ,\gamma ,\delta }(s+1)\frac{X^{s}}{s}\;ds\;dt.$$

Thus, adding the diagonal and off-diagonal terms we obtain that the conjecture for the correlations of values of *I*_*α*,*γ*_(*n*) also implies the conclusion of theorem 1.2.

## 4. Proof of theorem 1.1

First of all, we have

∑h=1∞rq(h)hA=∑h=1∞∑g∣qg∣hgμ(q/g)hA=∑g∣qg1−Aμ(qg)ζ(A)=q1−AΦ(1−A,q)ζ(A),

where

$$\Phi (x,q)=\prod_{p|q}\left(1-\frac{1}{p^{x}}\right).$$

Using this and the functional equation for *ζ*, we have to evaluate

$$\begin{array}{rl} & \frac{1}{{\left(2\pi i\right)}^{2}}\iint_{\begin{array}{c} |w-1|=\epsilon \\ |z-1|=\epsilon \end{array}}\sum_{q=1}^{\infty }q^{w+z-s-1}\Phi (w+z-s-1,q)\\ & \;\times \zeta (w+z-s-1)\sum_{m_{1}=1}^{\infty }\frac{I_{\alpha ,\gamma }(m_{1})e(m_{1}/q)}{m_{1}^{w}}\sum_{m_{2}=1}^{\infty }\frac{I_{\beta ,\delta }(m_{2})e(m_{2}/q)}{m_{2}^{z}}\;dw\;dz. \end{array}$$

We can identify the polar structure of the Dirichlet series here by passing to characters via the formula

e(mq)=∑d∣md∣q1ϕ(q/d)∑χmod(q/d)τ(χ¯)χ(md).

Assuming GRH, the only poles near *w*=1 arise from the principal characters $\chi_{q/d}^{\left(0\right)}$. Using

$$\tau (\chi_{q/d}^{\left(0\right)})=\mu \left(\frac{q}{d}\right),$$

we have that the poles of $\sum_{m=1}^{\infty }I_{\alpha ,\gamma }(m)e(m/q)m^{-w}$ are the same as the poles of

$$\begin{array}{rl} & \sum_{d|q}\frac{\mu (q/d)}{\phi (q/d)}\sum_{m=1}^{\infty }I_{\alpha ,\gamma }(md)\chi_{q/d}^{\left(0\right)}(m)m^{-w}d^{-w}\\ & \;=q^{-w}\sum_{d|q}\frac{\mu (d)}{\phi (d)}d^{w}\sum_{m=1}^{\infty }\frac{I_{\alpha ,\gamma }(mq/d)\chi_{d}^{\left(0\right)}(m)}{m^{w}} \end{array}$$

and the principal parts are the same. We replace $\chi_{d}^{\left(0\right)}(m)$ by ∑e∣de∣mμ(e). Thus, we have

$$q^{-w}\sum_{d|q}\frac{\mu (d)d^{w}}{\phi (d)}\sum_{e|d}\mu (e)e^{-w}\sum_{m=1}^{\infty }\frac{I_{\alpha ,\gamma }(meq/d)}{m^{w}}.$$

Now we need the polar structure of

$$\sum_{m=1}^{\infty }I_{\alpha ,\gamma }(mr)m^{-w}$$

for *r*=*qe*/*d*.

We use a lemma from [23] which asserts that if *A*(*w*)=*B*(*w*)*C*(*w*), where $A(w)=\sum_{m=1}^{\infty }$ (*a*(*m*)/*m*^*w*^), $B(w)=\sum_{m=1}^{\infty }(b(m)/m^{w})$ and $C(w)=\sum_{m=1}^{\infty }(c(m)/m^{w})$ then

$$\sum_{m=1}^{\infty }\frac{a(mr)}{m^{w}}=\sum_{r=r_{1}r_{2}}\sum_{m=1}^{\infty }\frac{b(mr_{1})}{m^{w}}\sum_{\begin{array}{c} m \end{array}=1(m,r_{1})=1}^{\infty }\frac{c(mr_{2})}{m^{w}}.$$

We apply this identity with *a*(*m*)=*I*_*α*,*γ*_(*m*), with *b*(*m*)=*m*^−*α*^ and with *c*(*m*)=*μ*(*m*)*m*^−*γ*^. Then

$$\sum_{m=1}^{\infty }\frac{b(mr_{1})}{m^{w}}=r_{1}^{-\alpha }\zeta (w+\alpha )$$

and

$$\sum_{(m,r_{1})=1}\frac{c(mr_{2})}{m^{w}}=\sum_{(m,r_{1})=1}\frac{\mu (mr_{2})}{m^{w+\gamma }r_{2}^{\gamma }}=\frac{\mu (r_{2})}{r_{2}^{\gamma }}\sum_{(m,r)=1}\mu (m)m^{-w-\gamma }=\frac{\mu (r_{2})r_{2}^{-\gamma }}{\Phi (w+\gamma ,r)\zeta (w+\gamma )}.$$

Now

$$\sum_{r=r_{1}r_{2}}\mu (r_{2})r_{1}^{-\alpha }r_{2}^{-\gamma }=r^{-\alpha }\sum_{r=r_{1}r_{2}}\mu (r_{2})r_{2}^{\alpha -\gamma }=r^{-\alpha }\Phi (\gamma -\alpha ,r).$$

Thus,

$$\sum_{m=1}^{\infty }\frac{I_{\alpha ,\gamma }(mr)}{m^{w}}=\frac{\zeta (w+\alpha )r^{-\alpha }\Phi (\gamma -\alpha ,r)}{\Phi (w+\gamma ,r)\zeta (w+\gamma )}.$$

In particular, we see that the only pole near to *w*=1 is at *w*=1−*α* with residue

$$\frac{r^{-\alpha }\Phi (\gamma -\alpha ,r)}{\Phi (1+\gamma -\alpha ,r)\zeta (1+\gamma -\alpha )}.$$

Inserting this with *r*=*qe*/*d* into the above, we now have that

$$\begin{array}{rl} {Res}_{w=1-\alpha }\sum_{m=1}^{\infty }\frac{I_{\alpha ,\gamma }(m)e(m/q)}{m^{w}} & =q^{\alpha -1}\sum_{d|q}\frac{\mu (d)d^{1-\alpha }}{\phi (d)}\sum_{e|d}\mu (e)e^{\alpha -1}\frac{{\left(qe/d\right)}^{-\alpha }\Phi (\gamma -\alpha ,qe/d)}{\Phi (1+\gamma -\alpha ,qe/d)\zeta (1+\gamma -\alpha )}\\ & =\frac{F_{\alpha ,\gamma }(q)}{q\zeta (1+\gamma -\alpha )}, \end{array}$$

where

$$F_{\alpha ,\gamma }(q)=q^{\alpha }\sum_{d|q}\frac{\mu (d)d^{1-\alpha }}{\phi (d)}\sum_{e|d}\mu (e)e^{\alpha -1}\frac{{\left(qe/d\right)}^{-\alpha }\Phi (\gamma -\alpha ,qe/d)}{\Phi (1+\gamma -\alpha ,qe/d)}$$

is a multiplicative function of *q*. At a prime *p*, we have

$$\begin{array}{rl} F_{\alpha ,\gamma }(p) & =p^{\alpha }\left(\frac{p^{-\alpha }\Phi (\gamma -\alpha ,p)}{\Phi (1+\gamma -\alpha ,p)}-\frac{p^{1-\alpha }}{p-1}\left(1-\frac{p^{\alpha -1}p^{-\alpha }\Phi (\gamma -\alpha ,p)}{\Phi (1+\gamma -\alpha ,p)}\right)\right)\\ & =\frac{\Phi (\gamma -\alpha ,p)}{\Phi (1+\gamma -\alpha ,p)}\left(1+\frac{1}{p-1}\right)-\frac{p}{p-1}\\ & =\frac{p}{\left(p-1\right)}\left(\frac{\Phi (\gamma -\alpha ,p)}{\Phi (1+\gamma -\alpha ,p)}-1\right)=\frac{p}{\left(p-1\right)}\left(\frac{\left(1-p^{\alpha -\gamma }\right)}{\left(1-p^{-1+\alpha -\gamma }\right)}-1\right)\\ & =\frac{p}{\left(p-1\right)}\frac{\left(-p^{\alpha -\gamma }+p^{-1+\alpha -\gamma }\right)}{\left(1-p^{-1+\alpha -\gamma }\right)}=\frac{-p^{\alpha -\gamma }}{\left(1-p^{-1+\alpha -\gamma }\right)}=-p^{\alpha -\gamma }+O\left(\frac{1}{p}\right). \end{array}$$

With *w*=1−*α* and *z*=1−*β*, we see that our sum is

$$\frac{\zeta (1-\alpha -\beta -s)}{\zeta (1-\alpha +\gamma )\zeta (1-\beta +\delta )}\sum_{q=1}^{\infty }q^{-1-\alpha -\beta -s}\Phi (1-\alpha -\beta -s,q)F_{\alpha ,\gamma }(q)F_{\beta ,\delta }(q).$$

Because of *F*_*α*,*γ*_(*p*)=−*p*^*α*−*γ*^+*O*(1/*p*), we have

$$\sum_{q=1}^{\infty }q^{-1-\alpha -\beta -s}\Phi (1-\alpha -\beta -s,q)F_{\alpha ,\gamma }(q)F_{\beta ,\delta }(q)=\zeta (1+\gamma +\delta +s)B_{\alpha ,\beta ,\gamma ,\delta }(s),$$

where *B* is an Euler product that is absolutely convergent for *s* near 0. We claim that *B*_*α*,*β*,*γ*,*δ*_(*s*)=*A*_−*β*,−*α*−*s*,*γ*+*s*,*δ*_. This is easily seen to be equivalent to showing that

$$B_{\alpha ,\beta ,\gamma ,\delta }(0)=A_{-\beta ,-\alpha ,\gamma ,\delta }.$$

To prove this, we first note that for *j*≥2 we have

$$\begin{array}{rl} F_{\alpha ,\gamma }(p^{j}) & =p^{j\alpha }\left(\frac{p^{-j\alpha }\Phi (\gamma -\alpha ,p)}{\Phi (1+\gamma -\alpha ,p)}-\frac{p^{1-\alpha }}{p-1}\left(\frac{p^{-(j-1)\alpha }\Phi (\gamma -\alpha ,p)}{\Phi (1+\gamma -\alpha ,p)}-p^{\alpha -1}\frac{p^{-\alpha j}\Phi (\gamma -\alpha ,p)}{\Phi (1+\gamma -\alpha ,p)}\right)\right)\\ & =\frac{\Phi (\gamma -\alpha ,p)}{\Phi (1+\gamma -\alpha ,p)}\left(1-\frac{p}{\left(p-1\right)}+p^{\alpha -1}\right)=\frac{\Phi (\gamma -\alpha ,p)}{\Phi (1+\gamma -\alpha ,p)}\left(-\frac{1}{\left(p-1\right)}+\frac{1}{\left(p-1\right)}\right)=0. \end{array}$$

Now the sum of the series

$$\sum_{j=0}^{\infty }p^{(-1-\alpha -\beta )j}\Phi (1-\alpha -\beta ,p^{j})F_{\alpha ,\gamma }(p^{j})F_{\beta ,\delta }(p^{j})$$

is just

$$\begin{array}{rl} & 1+p^{-1-\alpha -\beta }\Phi (1-\alpha -\beta ,p)F_{\alpha ,\gamma }(p)F_{\beta ,\delta }(p)\\ & \;=1+\frac{\left(1-1/p^{1-\alpha -\beta }\right)}{p^{1+\alpha +\beta }}\frac{p^{\alpha -\gamma }}{\left(1-p^{-1+\alpha -\gamma }\right)}\frac{p^{\beta -\delta }}{\left(1-p^{-1+\beta -\delta }\right)}\\ & \;=1+\frac{\left(1-1/p^{1-\alpha -\beta }\right)}{p^{1+\gamma +\delta }(1-p^{-1+\alpha -\gamma })(1-p^{-1+\beta -\delta })}\\ & \;={\left(1-\frac{1}{p^{1+\gamma +\delta }}\right)}^{-1}B_{\alpha ,\beta ,\gamma ,\delta }^{\left(p\right)}(0), \end{array}$$

where

$$B_{\alpha ,\beta ,\gamma ,\delta }^{\left(p\right)}(0)=\left(1-\frac{1}{p^{1+\gamma +\delta }}\right)\left(1+\frac{\left(1-1/p^{1-\alpha -\beta }\right)}{p^{1+\gamma +\delta }(1-p^{-1+\alpha -\gamma })(1-p^{-1+\beta -\delta })}\right).$$

The identity will be proven provided we can show that

$$1+\frac{\left(1-1/p^{1-\alpha -\beta }\right)}{p^{1+\gamma +\delta }(1-p^{-1+\alpha -\gamma })(1-p^{-1+\beta -\delta })}=\frac{\left(1-1/p^{1-\alpha +\gamma }-1/p^{1-\beta +\delta }+1/p^{1+\gamma +\delta }\right)}{\left(1-1/p^{1-\beta +\delta })(1-1/p^{1-\alpha +\gamma }\right)}.$$

This is equivalent to showing that

$$1+\frac{XCD(1-X/AB)}{\left(1-XC/A)(1-XD/B\right)}=\frac{\left(1-XC/A-XD/B+XCD\right)}{\left(1-XD/B)(1-XC/A\right)},$$

where *X*=1/*p*; *A*=*p*^−*α*^; *B*=*p*^−*β*^; *C*=*p*^−*γ*^; *D*=*p*^−*δ*^. This reduces to

$$\left(1-\frac{XC}{A}\right)\left(1-\frac{XD}{B}\right)+XCD\left(1-\frac{X}{AB}\right)=\left(1-\frac{XC}{A}-\frac{XD}{B}+XCD\right)$$

or

(A−XC)(B−XD)+XCD(AB−X)=AB−XC−XD+XABCD,

which is easily checked.

## 5. Conjecture 1

We can use the results of the previous two sections to formulate the conjecture that is part of the input for theorem 1.2.

We expect *I*_*α*,*γ*_(*n*)*I*_*β*,*δ*_(*n*+*h*) for *n* near *u* to behave on average like

$$\sum_{q=1}^{\infty }r_{q}(h)\frac{1}{{\left(2\pi i\right)}^{2}}\int_{|w-1|=\epsilon }\sum_{m=1}^{\infty }\frac{I_{\alpha ,\gamma }(m)e(m/q)}{m^{w}}u^{w-1}\;dw\int_{|z-1|=\epsilon }\sum_{n=1}^{\infty }\frac{I_{\beta ,\delta }(n)e(n/q)}{n^{z}}u^{z-1}\;dz.$$

The integrals over *w* and *z* are

$$\frac{F_{\alpha ,\gamma }(q)u^{-\alpha }}{q\zeta (1+\gamma -\alpha )}\;\frac{F_{\beta ,\delta }(q)u^{-\beta }}{q\zeta (1+\delta -\beta )},$$

respectively. Thus, *I*_*α*,*γ*_(*n*)*I*_*β*,*δ*_(*n*+*h*) behaves like

$$\frac{n^{-\alpha -\beta }}{\zeta (1+\gamma -\alpha )\zeta (1+\delta -\beta )}\sum_{q=1}^{\infty }\frac{r_{q}(h)F_{\alpha ,\gamma }(q)F_{\beta ,\delta }(q)}{q^{2}}.$$

In particular, we expect that

$$\sum_{n=1}^{\infty }\frac{I_{\alpha ,\gamma }(n)I_{\beta ,\delta }(n+h)}{n^{s}}-\frac{\zeta (s+\alpha +\beta )}{\zeta (1+\gamma -\alpha )\zeta (1+\delta -\beta )}\sum_{q=1}^{\infty }\frac{r_{q}(h)F_{\alpha ,\gamma }(q)F_{\beta ,\delta }(q)}{q^{2}}$$

is analytic in *σ*>*σ*_0_ for some *σ*_0_<1.

This leads us to the following conjecture.

**Conjecture 5.1**

There are numbers *ϕ*<1 and *ψ*>0 such that

$$\sum_{n\leq x}I_{\alpha ,\gamma }(n)I_{\beta ,\delta }(n+h)=m(x,h)+O(x^{\phi })$$

uniformly for *h*≪*x*^*ψ*^ where

$$m(x,h)=\frac{1}{\zeta (1+\gamma -\alpha )\zeta (1+\delta -\beta )}\sum_{q=1}^{\infty }\frac{r_{q}(h)F_{\alpha ,\gamma }(q)F_{\beta ,\delta }(q)}{q^{2}}\frac{x^{1-\alpha -\beta }}{1-\alpha -\beta }.$$

## 6. Conclusion

In subsequent papers, we will extend this process to averages of truncated ratios with any number of factors in the numerator and denominator.

## Appendix A

For ease of comparison with results in the literature, we give a more concrete expression for M.

First of all, we note that the Rankin–Selberg Dirichlet series has an Euler product

$$B_{\alpha ,\beta ,\gamma ,\delta }(s)=\sum_{m=1}^{\infty }\frac{I_{\alpha ,\gamma }(m)I_{\beta ,\delta }(m)}{m^{s}}=\prod_{p}\sum_{j=0}^{\infty }\frac{I_{\alpha ,\gamma }(p^{j})I_{\beta ,\delta }(p^{j})}{p^{js}}.$$

Now

$$\sum_{j=0}^{\infty }I_{\alpha ,\gamma }(p^{j})x^{j}=\frac{1-p^{-\gamma }x}{1-p^{-\alpha }x}=(1-p^{-\gamma }x)(1+p^{-\alpha }x+p^{-2\alpha }x^{2}+\cdots )$$

so that

$$I_{\alpha ,\gamma }(p^{j})=\left\{ \begin{array}{ll} p^{-\alpha j}(1-p^{\alpha -\gamma }) & \mathrm{if}\;j\geq 1\\ 1 & \mathrm{if}\;j=0. \end{array}\right.$$

Thus,

$$\begin{array}{rl} \sum_{j=0}^{\infty }I_{\alpha ,\gamma }(p^{j})I_{\beta ,\delta }(p^{j})x^{j} & =1+(1-p^{\alpha -\gamma })(1-p^{\beta -\delta })\sum_{j=1}^{\infty }p^{-(\alpha +\beta )j}x^{j}\\ & =\frac{1-p^{-\beta -\gamma }x-p^{-\alpha -\delta }x+p^{-\gamma -\delta }x}{1-p^{-\alpha -\beta }x} \end{array}$$

and

$$\begin{array}{rl} \sum_{m=1}^{\infty }\frac{I_{\alpha ,\gamma }(m)I_{\beta ,\delta }(m)}{m^{s}} & =\zeta (s+\alpha +\beta )\prod_{p}\left(1-\frac{1}{p^{s+\beta +\gamma }}-\frac{1}{p^{s+\alpha +\delta }}+\frac{1}{p^{s+\gamma +\delta }}\right)\\ & =\frac{\zeta (s+\alpha +\beta )\zeta (s+\gamma +\delta )}{\zeta (s+\alpha +\delta )\zeta (s+\beta +\gamma )}A_{\alpha ,\beta ,\gamma ,\delta }(s), \end{array}$$

where

$$A_{\alpha ,\beta ,\gamma ,\delta }(s)=\prod_{p}\frac{\left(1-1/p^{s+\gamma +\delta }\right)\left(1-1/p^{s+\beta +\gamma }-1/p^{s+\alpha +\delta }+1/p^{s+\gamma +\delta }\right)}{\left(1-1/p^{s+\beta +\gamma }\right)\left(1-1/p^{s+\alpha +\delta }\right)}.$$

Now it is an easy exercise to calculate that

Mα,β,γ,δ(T;X)=∫0∞ψ(tT)((t2π)−α−β(t2πX)γ+δζ(1+α+β)ζ(1+γ+δ)ζ(1+α+δ)ζ(1+β+γ)Aα,β,γ,δ(1)+(t2π)−α−βζ(1−β−α)ζ(1+γ+δ)ζ(1−β+δ)ζ(1−α+γ)A−β,−α,γ,δ(1)−X−γ−δ(γ+δ)ζ(1+α+β−γ−δ)ζ(1+α−γ)ζ(1+β−δ)Aα−γ−δ,β,−δ,δ(1)+(t2π)−α−β(t2πX)γ+δζ(1+γ+δ−α−β)ζ(1−α+γ)ζ(1−β+δ)(γ+δ)A−β,γ+δ−α,−δ,δ(1))dt+O(T1−η)

for some *η*>0.
